# Artificial intelligence-based comparison of the effects of duodenojejunostomy and sleeve gastrectomy on pancreatic morphology in Zucker diabetic fatty rats

**DOI:** 10.3389/fsurg.2026.1755398

**Published:** 2026-02-06

**Authors:** Luisa Schäfer, Ambrus Gabor Mályi, Jodok Fink, Gabriel Seifert, Mira Fink, Stephan Herrmann, Uwe Pohlen, Bernhard Hügel, Peter Bronsert, Goran Marjanovic, Stefan Fichtner-Feigl, Claudia Lässle

**Affiliations:** 1 Department of General and Visceral Surgery, Faculty of Medicine, Medical Center—University of Freiburg, University of Freiburg, Freiburg, Germany; 2 Faculty of Medicine, Institute for Surgical Pathology, Medical Center—University of Freiburg, University of Freiburg, Freiburg, Germany; 3 Department of General, Visceral and Vascular Surgery, Ortenau Klinikum Offenburg-Kehl, Offenburg, Germany; 4 EXCEL Excellent Clinician Scientist Program, Faculty of Medicine, Else Kroener Research Schools for Physicians, University of Freiburg, Freiburg, Germany

**Keywords:** AI-based tissue quantification, duodenojejunostomy, pancreatic morphology, sleeve gastrectomy, *β*-cell mass

## Abstract

**Objective:**

This study aims to compare the effects of sleeve gastrectomy (SG), duodenojejunostomy (DJOS), and their combination (DJOS + SG) on glucose regulation and pancreatic histomorphology and function in Zucker diabetic fatty (ZDF) rats, using artificial intelligence (AI)–assisted tissue analysis to assess morphological alterations.

**Methods:**

Forty-five male ZDF rats were randomized into three surgical groups (SG, DJOS, DJOS + SG). Oral glucose tolerance tests (OGTT) and insulin levels were assessed at 1, 3 and 6 months post-surgery. Pancreatic tissue was analyzed histologically and immunohistochemically for *β*-cell mass, PCNA and PDX-1 expression. QuPath software enabled AI-based quantification of acinar, adipose, and fibrotic tissue.

**Results:**

DJOS and DJOS + SG improved glucose tolerance and increased fasting insulin compared to SG. Both bypass groups demonstrated greater *β*-cell mass and clustering, elevated PCNA and PDX-1 expression, and more acinar tissue. SG was associated with reduced *β*-cell presence and increased pancreatic adiposity.

**Conclusion:**

Malabsorptive (DJOS) or combination bariatric procedures (DJOS + SG) significantly enhance glycemic control in the rat model. These effects are accompanied by increased *β*-cell numbers and clustering, as well as enhanced *β*-cell proliferation and differentiation. Furthermore, acinar glandular tissue is increased, while pancreatic adiposity is reduced following bypass surgery.

## 1 Introduction

Obesity, defined as a body mass index (BMI) > 30 kg/m^2^, is a growing, multifactorial condition with major implications for healthcare systems worldwide (1). Over the past four decades, the prevalence of obesity has tripled (1). Obesity is associated with numerous life-limiting comorbidities and plays a central role in the development of metabolic syndrome (2, 3).

When lifestyle modifications fail to achieve sufficient weight reduction, metabolic surgery has proven to be an effective therapeutic approach (4). Most patients maintain substantial weight loss and experience lasting improvement in obesity-related comorbidities (4). Two of the most commonly performed surgical procedures are Roux-en-Y gastric bypass (RYGB) and sleeve gastrectomy (SG) (5, 6). In addition to inducing significant weight loss, these procedures positively impact conditions such as hypertension, dyslipidemia, nonalcoholic fatty liver disease (NAFLD) or nonalcoholic steatohepatitis (NASH), sleep apnea and type 2 diabetes mellitus (T2D) (2, 3, 5).

In recent years, other combined restrictive-malabsorptive techniques—such as one-anastomosis gastric bypass (OAGB), single-anastomosis sleeve–ileal bypass (SASI), and single-anastomosis duodenal–ileal switch (SADI-S)—have gained popularity, yielding similarly favorable weight loss and glycemic control (5).

T2D is characterized by pancreatic *β*-cell dysfunction and reduced insulin sensitivity, leading to increased insulin demand (7). Its incidence has risen dramatically over the past decades (8–10). Approximately 80%–90% of patients with T2D are overweight or obese (8–10), and the risk of developing T2D increases with the degree of obesity (10). Weight loss improves glycemic control, and both RYGB and SG have been repeatedly compared in terms of weight reduction and remission of associated diseases (11–13). Overall, both procedures achieve similar outcomes (11–13). However, RYGB appears superior in long-term weight maintenance and discontinuation of oral antidiabetic medications (14–16), especially in patients with more severe or long-standing diabetes (17, 18). Notably, improvements in glycemic control, including normalization of glycated hemoglobin, often occur early after metabolic surgery, before significant weight loss is achieved, suggesting weight-independent metabolic effects (19, 20). The mechanisms underlying this rapid metabolic improvement remain incompletely understood.

In our previous publication, the metabolic effects of different surgical interventions were analyzed from a functional perspective, demonstrating early improvements in glucose tolerance and insulin secretion following bypass surgery (21). Building on these findings, the present study, applies a novel artificial intelligence (AI)-assisted histomorphological approach to examine pancreatic tissue in the same cohort of Zucker Diabetic Fatty (ZDF) rats, allowing a direct link between previously reported metabolic outcomes and tissue-level adaptations. Specifically, we compare duodenojejunostomy (DJOS), sleeve gastrectomy (SG), and the combined procedure (DJOS + SG), focusing on how these interventions differentially affect pancreatic structure, *β*-cell proliferation, differentiation, and overall histomorphology. This analysis aims to elucidate the mechanisms by which different surgical interventions influence pancreatic glucoregulation and histomorphology, thereby contributing to the distinct improvements in glycemic control observed after metabolic surgery.

By linking the previously reported functional outcomes with long-term tissue-level changes, this study provides mechanistic insight into how bariatric surgery preserves *β*-cell mass, enhances proliferation and differentiation, and modulates pancreatic exocrine tissue composition.

## 2 Material and methods

### 2.1 Diets and animals

Male obese Zucker diabetic fatty rats (ZDF- *lepr^fa^*/CRL) were obtained from Charles River Breeding Laboratories (Wilmington, MA, USA). The animals were 7 weeks old at the start of the experiment. Animal housing and feeding was performed as previously described (21). Rats were fasted for 4 h prior to surgery. A fasting period of 6 h was applied before the oral glucose tolerance test (OGTT) and hormone measurements. All experimental procedures were approved by the local animal welfare committee, and all applicable institutional and national guidelines for the care and use of animals were followed.

### 2.2 Experimental protocols

After purchase, rats were allowed to acclimatize for 14 days with free access to water and standard chow. Animals were then randomly assigned to one of the three operative groups, as previously described (21): sleeve gastrectomy (SG), duodenojejunostomy (DJOS), or duodenojejunostomy combined with sleeve gastrectomy (DJOS + SG). OGTTs were conducted 1, 3 and 6 months after surgery in all groups. Hormone measurements were obtained 2 days after each OGTT, 20 min following glucose stimulation. At the end of the study, animals were euthanized by lethal intracardial injection of potassium chloride (2 mmol/kg body weight) under general anesthesia. Following euthanasia, blood and tissue samples were collected for further analysis. Body weight was measured daily during the first postoperative week and then weekly thereafter. Water and food intake were monitored during the first postoperative week.

### 2.3 Surgery

SG and DJOS were performed as previously described (21). Briefly, SG was carried out via midline laparotomy (3–4 cm). The greater curvature of the stomach was exposed, and the gastrocolic and gastrosplenic ligaments were transected. Gastric resection was then performed using the Endo GIA^TM^ system (Universal Roticulator^TM^ 60-2.5Stapling System, Covidien), beginning approximately 5–8 mm proximal to the pylorus. The staple line was reinforced with PDS 6/0 sutures (Ethicon). For DJOS, the total length of the small intestine was measured after midline laparotomy. The duodenum was transected at its first portion, and the remaining duodenal stump was closed with PDS 6/0 sutures (Ethicon). A previously selected segment of the jejunum was then anastomosed to the duodenal stump in an end-to-side configuration, excluding the duodenum and the proximal one-third of the small intestine from the alimentary passage. Mesenteric defects were closed with PDS 6/0 sutures (Ethicon). In the DJOS + SG group, both procedures were performed sequentially. Anesthesia, perioperative analgesia and postoperative water and food management were conducted as previously described (21).

### 2.4 OGTT

OGTTs were performed under general anesthesia, as previously described (21). A 70% glucose solution (1 g/kg body weight; B. Braun, Melsungen, Germany) was administered via an orogastric tube (central venous catheter; Arrow Deutschland GmbH, Kernen, Germany). Blood glucose levels were measured from the tail vein at 0, 20, 60 and 120 min after glucose administration using a glucose meter (Accu-Check Aviva; Roche Diagnostics Deutschland GmbH, Mannheim, Germany).

### 2.5 Insulin measurement

Insulin measurements were performed as previously described by Laessle et al., using the same experimental setup as for the OGTT (21). Twenty minutes after glucose administration via the orogastric tube, 400 µL of blood were collected by cannulation of the tail vein. Collection tubes contained 0.69 mg K_3_EDTA (Sarstedt AG & Co, Nümbrecht, Germany). Samples were centrifuged at 5,000 rpm for 15 min at 4 °C, snap-frozen in liquid nitrogen, and then stored at −80 °C until further processing. Plasma insulin concentrations were determined by ELISA with horseradish peroxidase (HRP) reaction for detection (DRG Instruments GmbH, Marburg, Germany).

### 2.6 Immunostaining

Pancreatic tissue specimens were fixed in 4% paraformaldehyde solution and embedded in paraffin. Sections of 3 µm thickness were cut and stained for Pancreatic and Duodenal Homeobox 1 (PDX-1), Proliferating Cell Nuclear Antigen (PCNA) and hematoxylin and eosin (H&E). For PDX-1 and PCNA staining, sections were deparaffinized in Rotihistol (Roth, Karlsruhe, Germany). Antigen retrieval was performed in 10 mM citrate buffer, pH 6.0 (1.92 g citric acid monohydrate per 1 L dH_2_0, pH adjusted with NaOH; Merck; #244.1000). Endogenous peroxidase activity was blocked using Peroxidase-Blocking Solution (DAKO #S2023). Sections were then incubated with primary antibodies against PDX-1 (1:500; Abcam, #219.207, Cambridge, UK) and PCNA (1:30,000; Abcam, #29, Cambridge, UK). Antibody binding was visualized using the Dako EnVision system (K4003-HRP; Dako North America, Carpinteria, CA, USA) and developed with DAB + Substrate Chromogen System (DAKO, #K3468). Finally, sections were dehydrated in Rotihistol and mounted with Roti-Histokitt (Roth, Karlsruhe, Germany).

### 2.7 Digital assessment

Slides with conventional hematoxylin and eosin (H&E) and immunohistochemical staining were digitized using the VENTANA DP 200 slide scanner (Roche Diagnostics, Rotkreuz, Switzerland) at 20× magnification on a single focal plane. Each slide was independently reviewed by two pathologists prior to the digital analysis using QuPath (version 0.2.3). For conventional H&E slides, a threshold of 250 average RGB values was applied based on the mean red-green-blue (RGB) pixel intensity of each pixel. Pixels with values above this threshold were classified as a non-tissue areas, whereas those below were defined as tissue areas. When QuPath misclassified non-tissue areas (particularly adipose tissue), manual annotations were performed and verified by two pathologists. Regions of interest were manually classified into acinar, adipose, and fibrotic tissue types by two pathologists to generate a training dataset for the QuPath machine learning algorithm. This algorithm then classified the entire tissue area according to these predefined categories for each pancreatic section, allowing quantitative measurements of all tissue classes. For each section, a “self-adjusted” classifier was generated based on representative areas of adipose, glandular and fibrotic tissue from the same section.

Using QuPath's Smart Annotation Toolkit, pancreatic islets of Langerhans were annotated. Detection of positively stained cells was performed, and identified cells were classified as positive and negative according to the mean nuclear optical DAB density. Quantitative analyses included determining the absolute and relative numbers of positive and negative cells. For histological evaluation of pancreatic islets in the corpus and tail regions, five randomly selected visual fields per slide were examined at 5× magnification. For *β*-cell assessment, islet structures were categorized as either large islet cell clusters (defined as >10 *β*-cells) or isolated *β*-cells. Islet cell clusters were assigned a weighting factor of two, isolated cells a weighting factor of one, and the total number of *β*-cells was calculated as the weighted sum.

### 2.8 Statistical analyses

Statistical analyses were performed using GraphPad Prism 10 for macOS (GraphPad Software Inc., San Diego, CA, USA). Individual values were compared using the Mann–Whitney test. *P* values < 0.05 were considered statistically significant. The area under the curve (AUC) during the OGTT was calculated using GraphPad Prism 10.

## 3 Results

### 3.1 Study design and animal groups

**Figure 1:**
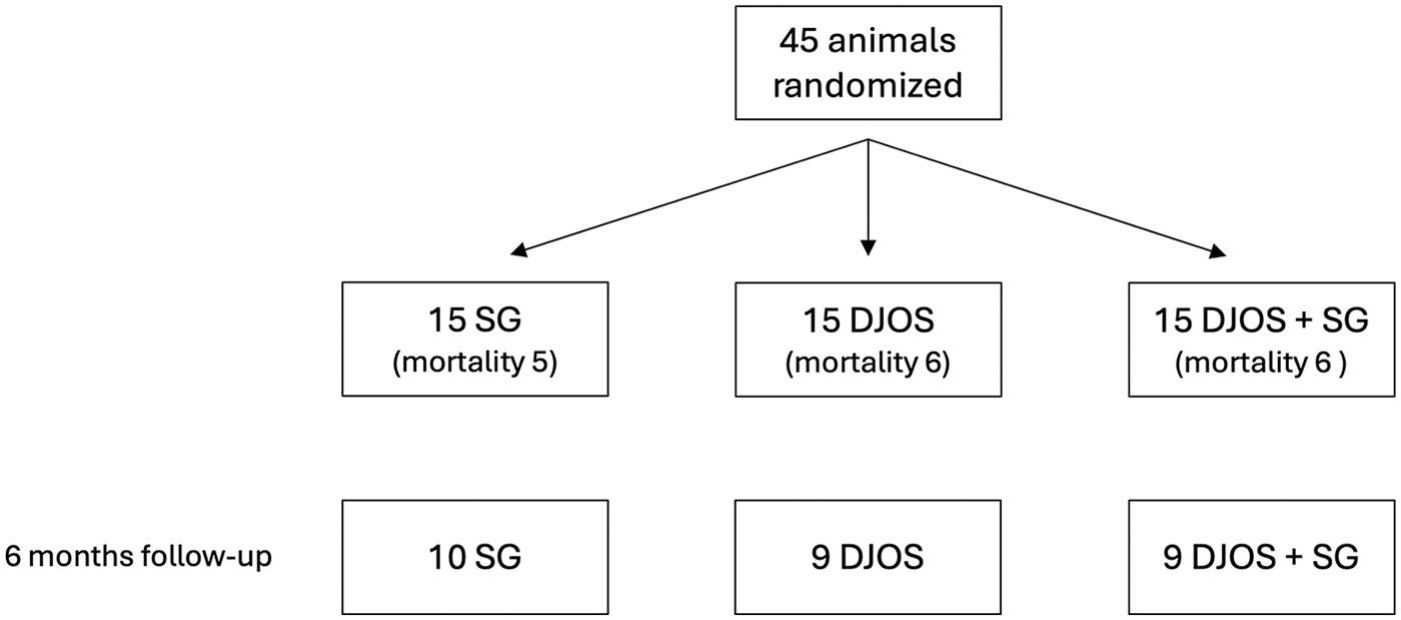
Flow chart depicting the number and mortality of zucker diabetic fatty (ZDF) rats throughout the entire experimental period, with an overall mortality rate of 37.8% (33.3% in the SG group and 40% in both the DJOS and DJOS + SG groups).

**Figure 2:**
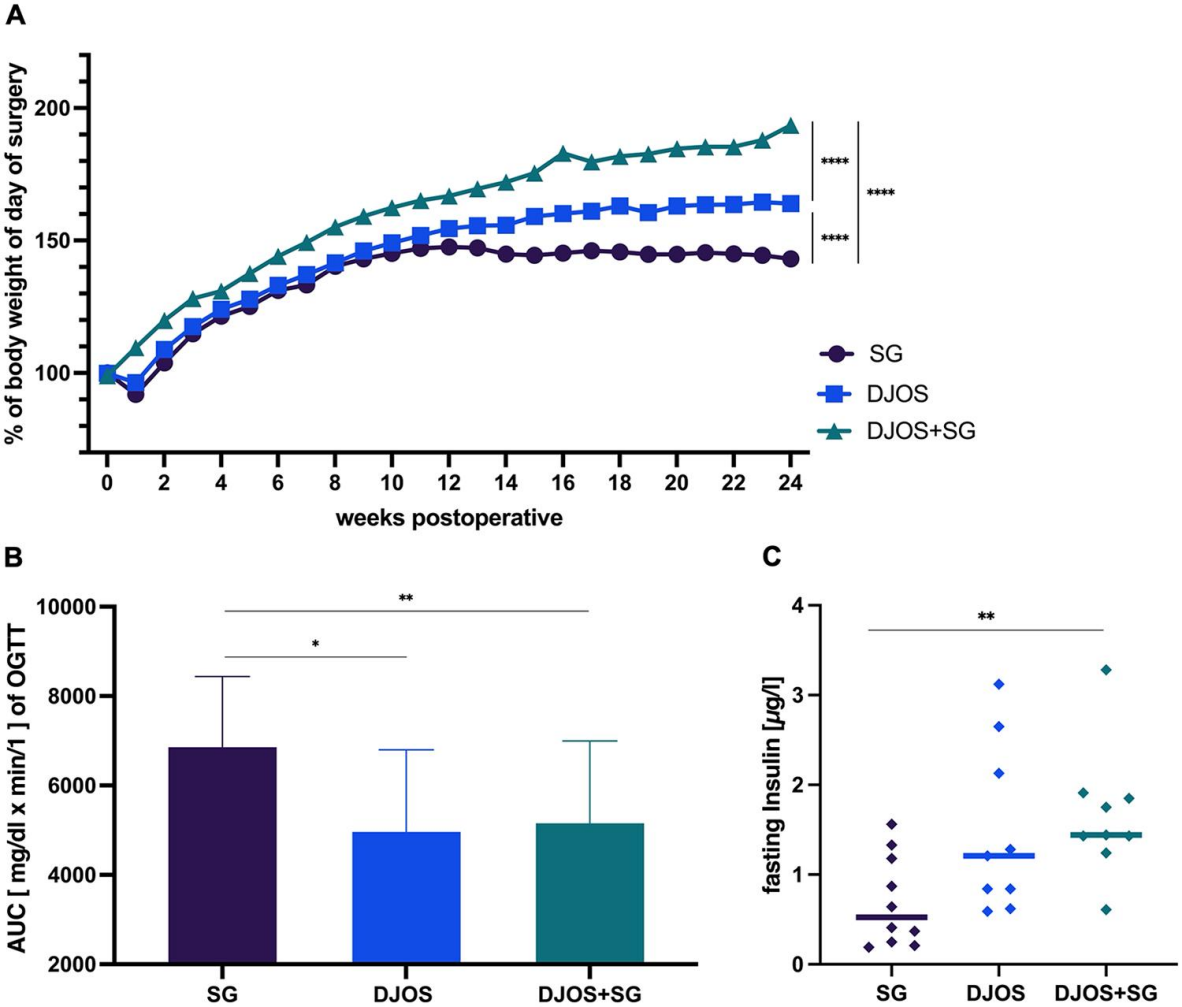
Overview of the metabolic status of the of zucker diabetic fatty (ZDF) rats. **(A)** Relative changes in body weight over the course of the experiment, normalized to 100% at baseline. **(B)** Area under the curve (AUC) of the oral glucose tolerance test (OGTT) 6 months postoperatively. **(C)** Fasting glucose levels measured 6 months postoperatively.

Figure 1 provides an overview of the study design and the allocation of animals to the respective groups. A total of 45 rats were randomized into three equally sized groups (*n* = 15 per group). The first group underwent a malabsorptive procedure via duodenojejunostomy (DJOS), the second group received a restrictive intervention through sleeve gastrectomy (SG), and the third group underwent a combination of both procedures (DJOS + SG). Postoperative weight changes are presented in Figure 2A. The overall mortality rate was 37.8% (33.3% in the SG group and 40% in both the DJOS and DJOS + SG groups), attributable to both postoperative mortality and the exceptionally long observation period. The extended follow-up represented a substantial strain, particularly for phenotypically compromised animals, and likely contributed to the increased mortality rate.

### 3.2 Glucoregulation and serum insulin

Changes in OGTT values were used to calculate the area under the curve (AUC). Six months after surgery, the total blood glucose increase during the OGTT was significantly higher in the SG group compared to DJOS and DJOS + SG (DJOS vs. SG, *p* = 0.0101; DJOS + SG vs. SG, *p* = 0.0076; DJOS vs. DJOS + SG, n.s.) (Figure 2B). Basal serum insulin levels were also higher in the DJOS and DJOS + SG groups compared to SG at 6 months postoperatively (Figure 2C). In particular, insulin levels in the DJOS + SG group were significantly elevated compared to SG (DJOS + SG vs. SG, *p* = 0.0027), whereas the SG group exhibited markedly reduced serum insulin concentrations.

### 3.3 Pancreatic *β*-cells

**Figure 3:**
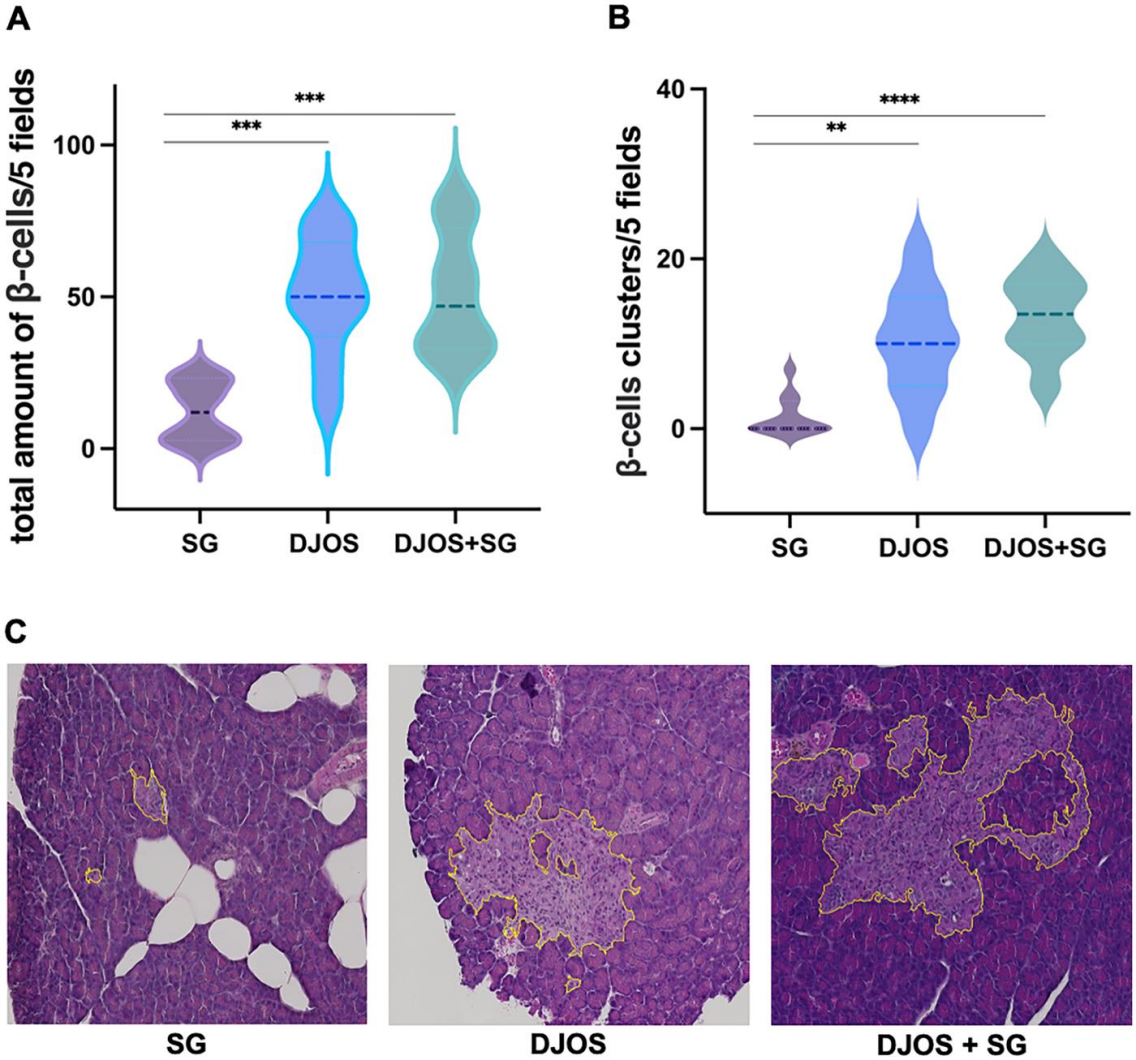
Histomorphological analysis of pancreatic tissue 6 months postoperatively. **(A)** Total number of pancreatic *β*-cells, quantified in five randomly selected visual fields, each examined at 5× magnification. **(B)** Number of *β*-cell clusters in five randomly selected visual fields, each examined at 5× magnification. **(C)** H&E staining of pancreatic tissue 6 months after sleeve gastrectomy (SG), duodenojejunostomy (DJOS), or duodenojejunostomy with sleeve gastrectomy (DJOS + SG). Pancreatic *β*-cells are outlined in yellow.

Both DJOS and DJOS + SG were associated with significantly higher numbers of insulin-producing *β*-cells compared to SG (DJOS vs. SG, *p* = 0.0004; DJOS + SG vs. SG, *p* < 0.0001; DJOS vs. DJOS + SG, n.s.) (Figure 3A). Similarly, the number of *β*-cell clusters was higher in the DJOS and DJOS + SG groups (Figure 3B). In contrast, pancreatic tissue samples from SG animals showed almost no *β*-cell clusters and, consequently, a significantly lower overall percentage of *β*-cells.

### 3.4 PCNA and PDX-1

**Figure 4:**
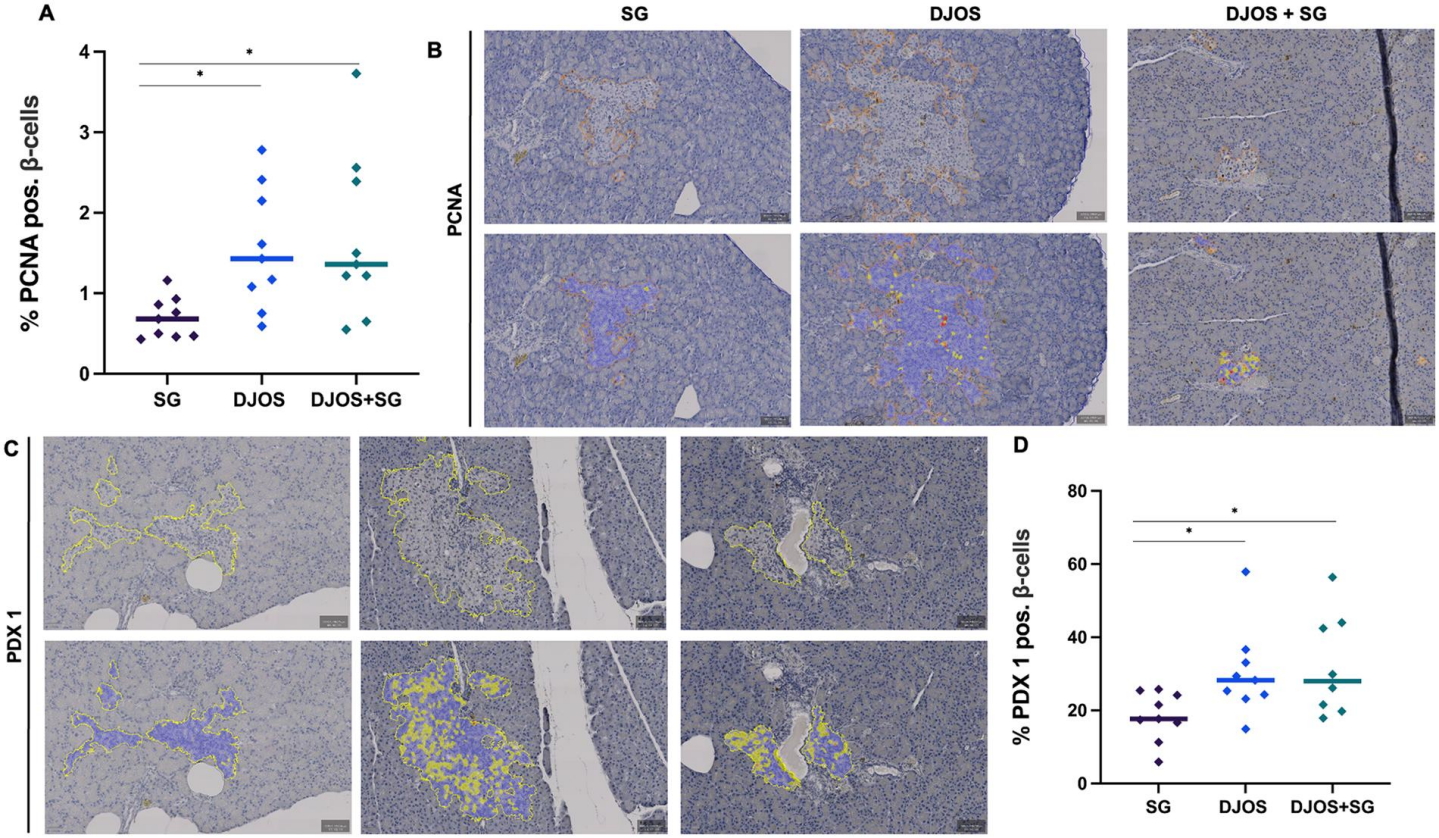
AI-assisted immunohistopathological analysis of *β*-cell proliferation and differentiation 6 months after sleeve gastrectomy (SG), duodenojejunostomy (DJOS), or duodenojejunostomy with sleeve gastrectomy (DJOS + SG). All analyses were performed at 20× magnification. **(A)** Percentage of PCNA-positive *β*-cells. **(B)** Representative graphs of the AI-based analysis of PCNA staining. **(C)** Representative graphs of the AI-based analysis regarding PDX-1 staining. **(D)** Percentage of PDX-1-positive *β*-cells.

DJOS and DJOS + SG were associated with significantly higher levels of PCNA expression compared to SG (DJOS vs. SG, *p* = 0.0056; DJOS + SG vs. SG, *p* = 0.0052; DJOS vs. DJOS + SG, n.s.) (Figures 4A,B). Similarly, PDX-1 expression was significantly higher in the DJOS and DJOS + SG groups than in SG (DJOS vs. SG, *p* = 0.0188; DJOS + SG vs. SG, *p* = 0.0152; DJOS vs. DJOS + SG, n.s.) (Figures 4C,D).

### 3.5 Quantitative pancreatic tissue analysis

**Figure 5:**
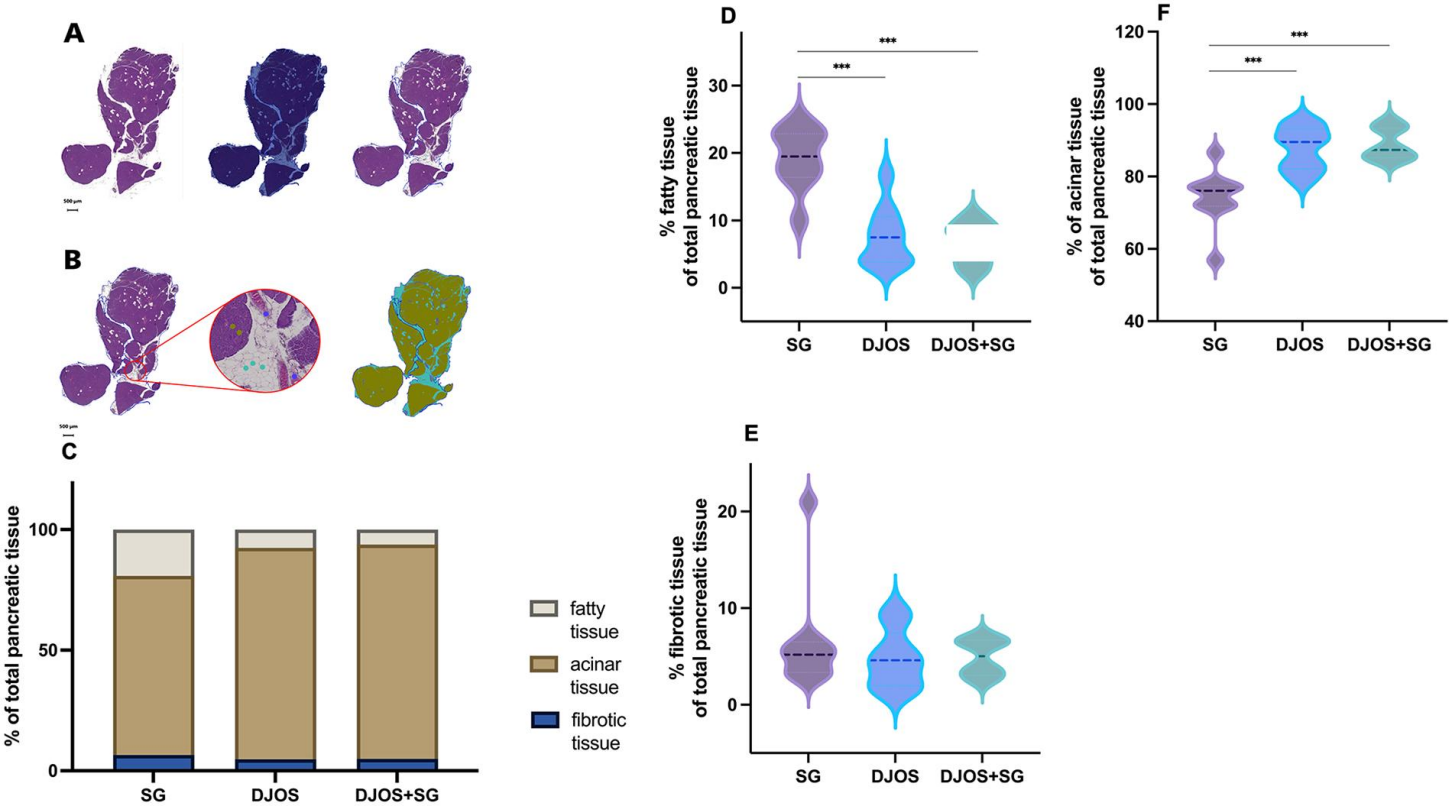
AI-assisted tissue recognition and classification of pancreatic histological structures. **(A)** Exemplary tissue recognition and classification workflow. Each digitized slide was analyzed by applying a default cutoff at the average RGB pixel value to distinguish tissue from non-tissue areas. **(B)** Representative regions of interest were annotated according to the predefined tissue types. The algorithm classified each histological structure as acinar (dark lime green circle), fibrotic (blue circle) or adipose tissue (cyan circle), enabling quantitative analysis of all tissue classes within each specimen. **(C)** Overview of absolute tissue composition. **(D)** Percentage of adipose tissue in the individual groups. **(E)** Percentage of fibrotic tissue in the individual groups. **(F)** Percentage of acinar tissue in the individual groups.

Quantitative histologic analyses revealed a significantly higher proportion of acinar tissue in DJOS and DJOS + SG compared to SG alone (DJOS vs. SG, *p* = 0.0005; DJOS + SG vs. SG, *p* = 0.0006) (Figures 5C,F). In contrast, fatty tissue was significantly more abundant in the SG group than in DJOS and DJOS + SG (DJOS vs. SG, *p* = 0.0003; DJOS + SG vs. SG, *p* = 0.0003; DJOS vs. DJOS + SG, n.s.) (Figures 5C,D). No significant differences were observed between groups with respect to stromal tissue content (Figures 5C,E).

## 4 Discussion

Bariatric surgery provides substantial benefits in terms of weight loss and remission of obesity-related comorbidities, particularly type 2 diabetes mellitus (T2D) (2, 22–25). In this study, we used Zucker Diabetic Fatty (ZDF) rats, a well-established monogenic model for T2D, which develops severe obesity and diabetes (26). It is important to note that this model primarily reflects a single-gene defect, whereas the human T2D condition is multifactorial and influenced by genetic, environmental, and lifestyle factors. A hallmark of ZDF rats is *β*-cell loss during adolescence, leading to the rapid onset of severe diabetes (26, 27).

In clinical practice, treatment strategies are increasingly shifting away from a “one-procedure-fits-all” paradigm towards more personalized approaches. Determining the optimal surgical procedure for individual patients remains a key challenge. Various recommendations have been proposed in the literature, including the use of bariatric surgery calculators to aid decision-making (28). In most cases, the initial choice is between sleeve gastrectomy (SG) and Roux-en-Y gastric bypass (RYGB) (15).

Several studies and meta-analyses have demonstrated that bariatric surgery provides superior long-term glycemic control in patients with T2D compared to drug therapy alone (2, 24, 25). However, despite initial diabetes remission, some patients experience relapse over time (25, 29). In the short term, SG has been shown to be as effective as RYGB in achieving glycemic control (11, 20, 30–33). The long-term superiority of RYGB over SG remains inconclusive, with studies reporting mixed results (11, 15, 34–39). Nevertheless, there is evidence suggesting that RYGB may confer to a potential advantage regarding sustained antidiabetic effects.

To investigate mechanisms underlying these outcomes, we employed a rat model exhibiting an extreme diabetic phenotype. Ob/ob rats, which carry a leptin receptor mutation, develop severe T2D during adolescence, eventually leading to complete endocrine pancreatic insufficiency (40). The SG group underwent substantial stomach resection to produce a restrictive effect, analogous to the human procedure. DJOS involved duodenal und proximal jejunal exclusion, analogous to human SADI-S (single anastomosis duodeno-ileal bypass with sleeve gastrectomy) or OAGB (one-anastomosis gastric bypass), without stomach resection. The combination procedure (DJOS + SG) added a restrictive component.

Importantly, the animals analyzed here represent the same cohort used in our previous publication comparing SG, DJOS, and DJOS + SG (21). Early postoperatively, all groups exhibited similar glucose tolerance; however, at 3 and 6 months, the bypass groups demonstrated superior glycemic outcomes (21). Fasting insulin levels measured six months postoperatively—a substantial timeframe for ZDF rats—similarly indicated that the bypass groups (DJOS and DJOS + SG) achieved significantly better glycemic outcomes than SG alone (Figures 2A–C) (21). These results are consistent with clinical data: McTique et al. reported that five years after RYGB, patients exhibited slightly higher T2D remission rates, fewer relapses and improved long-term glycemic control compared to SG (15).

Building on these previously published data, the current analyses extend the findings to long-term pancreatic morphology, *β*-cell proliferation, differentiation, and tissue composition, providing mechanistic insights that were not included in the prior study.

Given the natural *β*-cell loss in our model, we investigated pancreatic changes following bypass procedures. Microscopic examination revealed *β*-cell clustering in the bypass groups (DJOS and DJOS + SG), whereas SG animals predominantly exhibited solitary *β*-cells. These observations align with rodent studies showing RYGB-induced *β*-cell mass (41–43). Human studies similarly report improved *β*-cell function after bariatric surgery, as indicated by fasting glucose and insulin values, disposition index (DI), proinsulin-to-C-peptide ratio, and insulin secretion relative to glucose (44–47). Short-term outcomes, however, do not differ significantly between RYGB and SG, suggesting that mechanisms conferring RYGB's long-term advantages manifest over time (44, 46, 47). Our previously published data confirm an early glycemic benefit in SG animals that diminishes in the long term (21).

Proliferating Cell Nuclear Antigen (PCNA) and PDX-1 were used to assess *β*-cell proliferation and differentiation. PCNA reflects regenerative capacity, while PDX-1 is essential for *β*-cell function, including insulin gene expression and secretion; downregulation can lead to *β*-cell dedifferentiation (48, 49). Talchai et al. propose that the loss of *β*-cell function in T2D is due to dedifferentiation, accompanied by a decrease in maturation markers (50, 51). Notably, bariatric procedures can reverse dedifferentiation and enhance *β*-cell identity and functionality (52–54). In our study, bypass groups exhibited higher *β*-cell mass, increased proliferation markers, and elevated PDX-1 levels compared to SG alone (Figure 4D). Li et al. similarly describe a marked rise in PDX-1 levels at mRNA and protein levels in diabetic rats following RYGB surgery peaking two weeks postoperatively (55). Importantly, the SG procedure in the DJOS + SG group did not provide additional metabolic benefits, suggesting that the bypass itself drives *β*-cell proliferation and long-term antidiabetic effects.

Quantitative AI-based tissue analysis revealed distinct fatty degeneration and loss of acinar cells in the SG group compared to the bypass groups (Figure 5C). Pancreatic steatosis is commonly associated with obesity and metabolic disorders (56–58), and bariatric surgery has been shown to reduce pancreatic fat deposits in both rodents and humans (58, 59). Notably, Salman et al. report a positive correlation between weight loss and the reduced pancreatic fat volume following SG (58, 59). Our findings indicate that different bariatric procedures may differentially affect pancreatic exocrine tissue, with bypass surgery preserving acinar tissue and reducing fatty infiltration.

Interestingly, across all measures examined—including glucose tolerance, fasting insulin levels, markers of *β*-cell proliferation and function, as well as histological assessment of pancreatic steatosis and acinar tissue—the addition of a restrictive component in DJOS + SG did not confer any further benefit over DJOS alone. This suggests that the long-term metabolic improvements appear to be predominantly driven by duodenal exclusion, potentially reflecting a ceiling effect in hormonal adaptation or the dominant influence of malabsorptive, hormone-mediated mechanisms.

A limitation of this study is the restricted translatability of the animal model, given anatomical differences such as an intact pylorus and relatively large gastric volume. The model was designed to compare metabolic consequences of duodenal bypass vs. sleeve gastrectomy, rather than to replicate a specific human procedure. Additionally, the ZDF rat represents a monogenic model of T2D, whereas the disease in bariatric patients is typically multifactorial. Nonetheless, the metabolic effects observed provide valuable mechanistic insights.

Overall, these results indicate that bariatric surgery—particularly bypass procedures—beneficially impacts long-term pancreatic morphology and function. In ZDF rats, where *β*-cells typically undergo programmed cell death during adolescence, bypass surgery preserved *β*-cell proliferation and clustering even after six months, suggesting a protective effect against *β*-cell apoptosis.

By using the same cohort of animals previously reported by Laessle et al. (21), we demonstrated that early improvements in glycemic control are maintained long-term and are accompanied by sustained enhancements in *β*-cell mass, proliferation, differentiation, and pancreatic tissue composition.

Although the model cannot fully replicate human T2D, these findings support clinical observations that bypass surgery offers superior metabolic benefits at the *β*-cell level. These findings align with clinical evidence from McTigue et al., demonstrating that bariatric bypass surgery provides superior outcomes for T2D compared to sleeve gastrectomy (15). However, it's important to note that specific patient groups—such as those with early-stage diabetes—can benefit equally from sleeve procedures, as shown in Aminian et al., highlighting the need for individualized treatment strategies (28).

## 5 Conclusion

Our data demonstrate that alterations of gastrointestinal anatomy via malabsorptive or combined bariatric surgery significantly improve glycemic control in a T2D rat model. These effects are associated with preservation of *β*-cells, increased *β*-cell number and clustering, enhanced proliferation and differentiation, and histological improvements in pancreatic tissue, including increased acinar content and reduced fatty infiltration. Importantly, the animals analyzed in this study are the same cohort previously published by Laessle et al. (21), in which early metabolic improvements were reported. The current analyses extend those findings by showing that these long-term metabolic benefits are accompanied by sustained enhancements in *β*-cell morphology, proliferation, differentiation, and exocrine pancreatic tissue composition, providing mechanistic insight into how bariatric surgery exerts durable antidiabetic effects.

## Data Availability

The raw data supporting the conclusions of this article will be made available by the authors, without undue reservation.
